# Clinical Trial Data Review of the Combination FTD/TPI + Bevacizumab in the Treatment Landscape of Unresectable Metastatic Colorectal Cancer

**DOI:** 10.1007/s11864-024-01261-w

**Published:** 2024-09-26

**Authors:** Thierry André, Eric Van Cutsem, Julien Taieb, Marwan Fakih, Gerald W. Prager, Fortunato Ciardiello, Alfredo Falcone, Mark Saunders, Nadia Amellal, Lucas Roby, Josep Tabernero, Per Pfeiffer

**Affiliations:** 1 Department of Medical Oncology, Saint-Antoine Hospital, AP-HP, INSERM 938, SIRIC CURAMUS, Sorbonne University, 184 Rue du Faubourg Saint Antoine, 75012 Paris, France; 2 Digestive Oncology, University Hospitals Gasthuisberg Leuven, KU Leuven, Leuven, Belgium; 3 Department of Gastroenterology and Digestive Oncology, Georges Pompidou European Hospital, SIRIC CARPEM, Université Paris-Cité, Paris, France; 4 City of Hope Helford Clinical Research Hospital, Duarte, CA USA; 5 Department of Medicine I, AKH Wien, Medical University of Vienna, Vienna, Austria; 6 Division of Medical Oncology, Department of Precision Medicine, University of Campania Luigi Vanvitelli, Naples, Italy; 7 University Hospital of Pisa, Pisa, Italy; 8 The Christie Hospital, Manchester, UK; 9 Servier International Research Institute, Suresnes, France; 10 Vall d’Hebron Hospital Campus and Institute of Oncology (VHIO), UVic-UCC, Barcelona, Spain; 11 Department of Oncology, Odense University Hospital, Odense, Denmark

**Keywords:** Metastatic colorectal cancer, FTD/TPI, Bevacizumab, Efficacy, Combination therapy

## Abstract

Recommended first and second line treatments for unresectable metastatic colorectal cancer (mCRC) include fluorouracil-based chemotherapy, anti-vascular endothelial growth factor (VEGF)-based therapy, and anti-epidermal growth factor receptor-targeted therapies. In third line, the SUNLIGHT trial showed that trifluridine*/*tipiracil + bevacizumab (FTD/TPI + BEV) provided significant survival benefits and as such is now a recommended third line regimen in patients with refractory mCRC, irrespective of *RAS* mutational status and previous anti-VEGF treatment. Some patients are not candidates for intensive combination chemotherapy as first-line therapy due to age, low tumor burden, performance status and/or comorbidities. Capecitabine (CAP) + BEV is recommended in these patients. In the SOLSTICE trial, FTD/TPI + BEV as a first line regimen in patients not eligible for intensive therapy was not superior to CAP + BEV in terms of progression-free survival (PFS). However, in SOLSTICE, FTD/TPI + BEV resulted in similar PFS, overall survival, and maintenance of quality of life as CAP + BEV, with a different safety profile. FTD/TPI + BEV offers a possible first line alternative in patients for whom CAP + BEV is an unsuitable treatment. This narrative review explores and summarizes the clinical trial data on FTD/TPI + BEV.

## Introduction

Unless patients with metastatic colorectal cancer (mCRC) are microsatellite instability high, they generally receive a first and second line regimen of treatment with fluorouracil-based chemotherapy (in combination with oxaliplatin and/or irinotecan), anti-vascular endothelial growth factor (anti-VEGF)-based therapy (mainly bevacizumab [BEV]), and anti-epidermal growth factor receptor (anti-EGFR)-targeted therapies (e.g., in patients with *RAS* and *BRAF* wild-type tumors in left colon, or specifically, anti-EGFR with encorafenib for patients with *BRAF* V600E mutated tumors) [1, 2]. However, some patients with mCRC may not be candidates for intensive full-dose doublet or triplet chemotherapy as an initial treatment regimen for a variety of reasons, including advanced age, poor performance status, comorbidities, low tumor burden or patient preference [1, 3]. In these patients, the recommended initial treatment regimen is fluoropyrimidine-based therapy plus BEV [1], usually the combination of capecitabine plus BEV (CAP + BEV) [4].

Patients who have disease progression after receiving these therapies are considered to have refractory disease [5]. When patients have a Eastern Cooperative Oncology Group performance status (ECOG PS) score < 2 they are eligible for further treatments [5] that include reintroduction of chemotherapeutic agents such as irinotecan, rechallenge with anti-EGFR therapy in patients with *RAS* wildtype disease [6, 7], or alternative chemotherapy regimens with or without angiogenesis inhibitors (such as BEV) [1]. Trifluridine*/*tipiracil (FTD/TPI) and regorafenib are approved therapies for patients who have progressed through all standard therapies. As a result of the survival benefits in the SUNLIGHT trial, FTD/TPI + BEV is now a recommended third line regimen in patients with mCRC [2, 8, 9]. The aim of this narrative review is to review the clinical trial data on the combination of FTD/TPI + BEV as an initial or later line regimen in mCRC, with a focus on efficacy. Safety of FTD/TPI + BEV will be reported in a separate review.

## Rationale for Combining FTD/TPI + BEV

FTD/TPI (also known as TAS-102) has shown OS benefit in patients with mCRC refractory or intolerant to a wide range of previous treatments including fluoropyrimidine, irinotecan, oxaliplatin, anti-VEGF, or anti-EGFR [10, 11]. The combination of chemotherapeutic agents and/or molecularly targeted agents can provide additive or synergistic effects in oncology. For example, CAP + BEV (for patients not suitable for intensive full-dose doublet or triplet chemotherapy) has lengthened survival time in patients with mCRC compared with monotherapy [4]. Similarly, combining FTD/TPI with other anticancer agents has the potential to enhance its efficacy, with preclinical data showing the potential for additive synergistic effects of FTD/TPI in combination with other classes of agents, such as anti-angiogenic therapies (nintedanib), EGFR inhibitors (cetuximab, panitumumab), and chemotherapies (irinotecan, oxaliplatin) [12].

Because BEV had beneficial effects in mCRC in combination with fluoropyrimidine (5-FU or CAP) based chemotherapy without overlapping toxicity [4, 13, 14], the combination of FTD/TPI + BEV was assessed for beneficial effects. Preclinical studies showed that in CRC xenografts, inhibition of tumor growth was significantly enhanced with FTD/TPI + BEV compared with either agent alone and that phosphorylated FTD levels were increased by combining FTD/TPI and BEV [15]. This suggests that BEV may help increase FTD accumulation and its subsequent phosphorylation in tumors by normalizing tumor vasculature [15]. This provided the rationale for subsequent clinical trials of FTD/TPI + BEV, described below, and this review will focus on efficacy data and what this means for clinical practice. Basic study design information on five key trials, C-TASK FORCE, TASCO-1, the Danish trial, SUNLIGHT and SOLSTICE, is presented in Table 1.

**Table 1:** Study design of 5 key FTD/TPI + BEV trials [8, 16–19]

	C-TASK FORCE	TASCO-1	Danish trial	SOLSTICE	SUNLIGHT
Phase	I/II	II	II	III	III
Inclusion criteria	Patients with unresectable mCRC				
	Patients refractory or intolerant to fluoropyrimidine, irinotecan, oxaliplatin, anti-VEGF, anti-EGFR	Untreated patients, not eligible for intensive therapy	Patients refractory or intolerant to fluoropyrimidine, irinotecan, oxaliplatin, anti-VEGF, anti-EGFR	Untreated patients, not eligible for intensive therapy	Patients refractory or intolerant to fluoropyrimidine, irinotecan, oxaliplatin, anti-VEGF, anti-EGFR
Treatment regimen	≥ 3	1	≥ 3	1	3
Experimental arm	FTD/TPI + BEV				
Comparator arm	None	CAP + BEV	FTD/TPI	CAP + BEV	FTD/TPI
Primary objective	Phase I: Safety Phase II: PFS at 16 weeks	PFS	PFS	PFS	OS
Secondary objectives	PFS, OS, PK	OS, QoL, safety	OS, response, safety	OS, safety, QoL	PFS, QoL, safety

Abbreviations: *BEV* bevacizumab, *CAP* capecitabine, *EGFR* epidermal growth factor receptor, *FTD/TPI* trifluridine/tipiracil, *mCRC* metastatic colorectal cancer, *OS* overall survival, *PFS* progression free survival, *PK* pharmacokinetics, *QoL* quality of life, *VEGF* vascular endothelial growth factor

## Clinical Data on FTD/TPI + BEV in Patients with Refractory mCRC (Third Line)

Key efficacy data regarding FTD/TPI + BEV as a third line regimen are presented in Table 2.

**Table 2:** Efficacy results of key trials with FTD/TPI + BEV in patients with refractory mCRC (third line) and in the first line setting [8, 16–21]

	Third line setting					First line setting			
	C-TASK FORCE	Danish trial		SUNLIGHT		TASCO-1		SOLSTICE	
Treatment arm	FTD/TPI + BEV	FTD/TPI + BEV	FTD/TPI	FTD/TPI + BEV	FTD/TPI	FTD/TPI + BEV	CAP + BEV	FTD/TPI + BEV	CAP + BEV
Number of patients	25	46	47	246	246	77	76	426	430
OS, median, months (95% CI)	11.4 (7.6–13.9)	9.4 (7.6–10.7)	6.7 (4.9–7.6)	10.8 (9.4–11.8)	7.5 (6.3–8.6)	22.3 (18.0–23.7)	17.7 (12.6–19.8)	19.7 (18.0–22.4)	18.6 (16.8–21.4)
PFS, median, months (95% CI)	3.7 (2.0–5.4)	4.6 (3.5–6.5)	2.6* (1.6–3.5)	5.6 (4.5–5.9)	2.4 (2.1–3.2)	9.2 (7.6–11.6)	7.8 (5.5–10.1)	9.4 (9.1–10.9)	9.3 (8.9–9.8)
CR, n (%)	0 (0)	0 (0)	0 (0)	0 (0)	1 (0)	0 (0)	0 (0)	6 (1)	3 (1)
PR, n (%)	0 (0)	1 (2)	0 (0)	15 (6)	2 (1)	26 (34)	23 (30)	147 (35)	176 (41)
SD, n (%)	16 (64)	30 (65)	24 (51)	NR	NR	40 (52)	36 (47)	215 (50)	187 (43)
ORR, n (%)	0 (0)	1 (2)	0 (0)	15 (6)	3 (1)	26 (34)	23 (30)	153 (36)	179 (42)
DCR, n (%)	16 (64)	31 (67)	24 (51)	NR	NR	66 (86)	59 (78)	368 (86)	366 (85)

^*^Investigator assessed; Abbreviations: *BEV* bevacizumab, *CAP* capecitabine, *CR* complete response, *DCR* disease control rate, *FTD/TPI* trifluridine/tipiracil, *mCRC* metastatic colorectal cancer, *NR* not reported, *ORR* objective response rate, *OS* overall survival, *PFS* progression free survival, *PR* partial response, *SD* stable disease

### Early Phase Studies

The efficacy and safety of FTD/TPI + BEV was first evaluated in a phase 1/2 trial in Japan in patients refractory or intolerant to fluoropyrimidine, irinotecan, oxaliplatin, anti-VEGF, or anti-EGFR (C-TASK FORCE) [16]. FTD/TPI + BEV was tested as a third or later line regimen in 25 patients. Dosage of FTD/TPI was 35 mg/m^2^ of body surface area, given orally twice a day on days 1–5 and 8–12 in a 28-day cycle, plus BEV (5 mg/kg of bodyweight, administered by intravenous infusion for 30 min every 2 weeks) and no dose-limiting toxicities were observed, which established this dosage as the recommended phase 2 dose. Centrally assessed progression-free survival (PFS) at 16 weeks was 42.9% (80% CI 27.8–59.0), exceeding the prespecified threshold based on FTD/TPI alone. In addition, centrally assessed disease control rate (DCR) was 64.0%, showing that the combination of FTD/TPI + BEV had significant clinical activity. Median overall survival (mOS) was 11.4 months in C-TASK FORCE [16], which was promising in comparison to the pivotal FTD/TPI trial RECOURSE [10], where mOS was 7.1 months, and hence led to the planning of further clinical trials.

A phase 2 trial carried out in Denmark in patients with mCRC refractory or intolerant to fluoropyrimidines, irinotecan, oxaliplatin, anti-VEGF or anti-EGFR, aimed to determine if FTD/TPI + BEV significantly prolonged PFS and OS compared to FTD/TPI [17]. In the trial, 47 patients received FTD/TPI and 46 received FTD/TPI + BEV. Median PFS was significantly improved in patients receiving FTD/TPI + BEV compared with patients receiving FTD/TPI (HR 0.45, 95% CI 0.29–0.72; *p* = 0.001) as was mOS (HR 0.55, 95% CI 0.32–0.94; *p* = 0.028; Table 2). Exploratory post-hoc subgroup analyses of OS favored FTD/TPI + BEV compared with FTD/TPI in most subgroups, including in the *RAS* mutant subgroup (HR 0.38, 95% CI 0.19–0.79), in patients ≥ 70 years old (HR 0.42, 95% CI 0.13–1.30), and in patients who had received previous BEV (HR 0.61, 95% CI 0.35–1.10) [17]. Similar results were observed for post-hoc subgroup analyses of PFS [17]. These results suggested that further investigation into safety and efficacy of FTD/TPI + BEV compared to FTD/TPI was worthwhile in larger-scale trials.

### The Pivotal Phase 3 Trial, SUNLIGHT

**Fig. 1:**
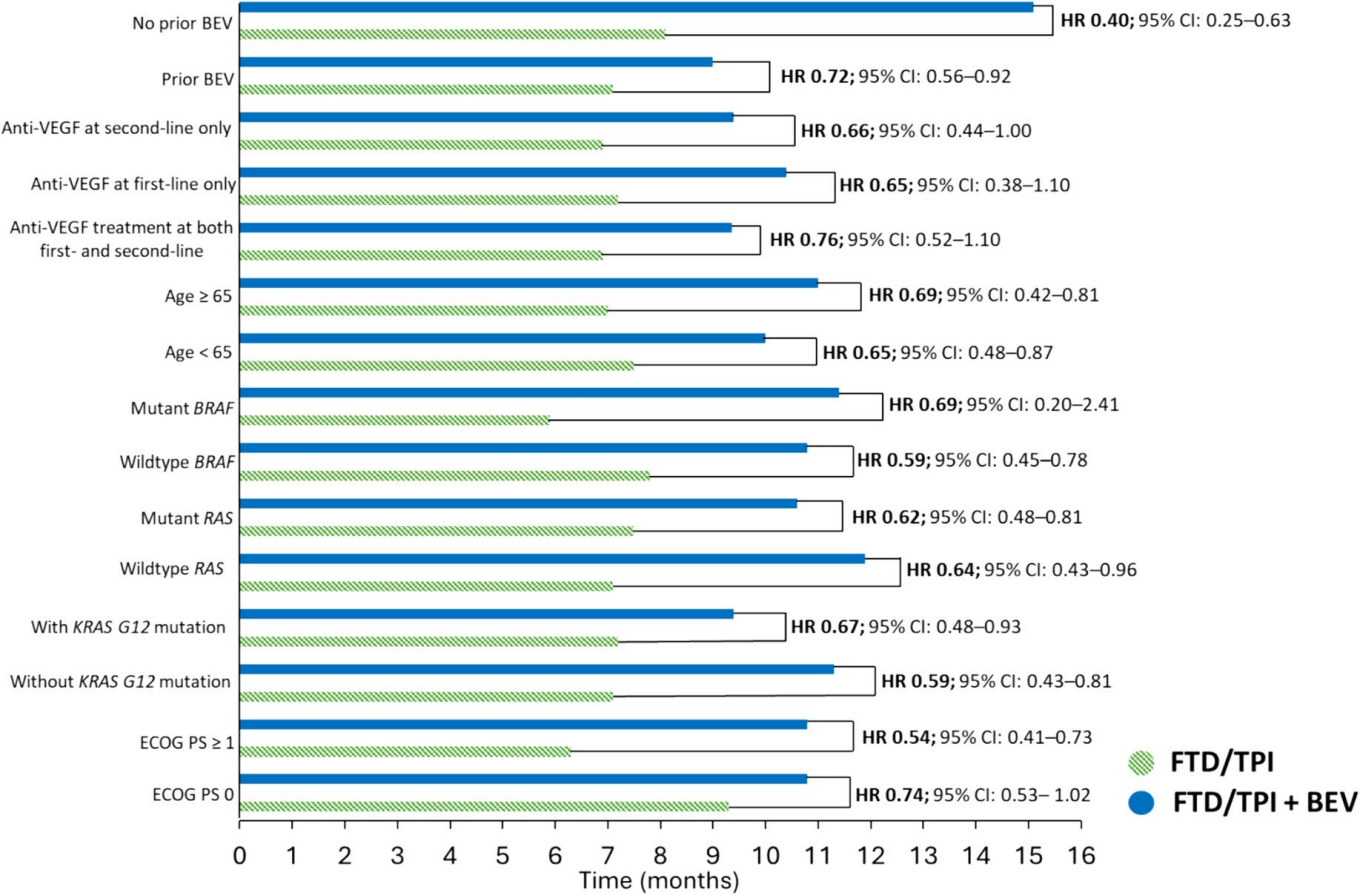
Median overall survival in subgroups in the SUNLIGHT trial [23, 26]. Abbreviations: BEV, bevacizumab; CI, confidence interval; ECOG, Eastern Cooperative Oncology Group; FTD/TPI, trifluridine/tipiracil; HR, hazard ratio; VEGF, vascular endothelial growth factor

The phase 3 international trial SUNLIGHT was designed with similar inclusion criteria for patients as the C-TASK FORCE and Danish trials [16, 17]. In the SUNLIGHT trial, 246 patients received FTD/TPI and 246 received FTD/TPI + BEV [8]. Median OS was significantly improved in patients receiving FTD/TPI + BEV compared with patients receiving FTD/TPI (HR 0.61, 95% CI 0.49–0.77; *p* < 0.001; Table 2). OS at 12 months was 43% in the FTD/TPI + BEV group and 30% in the FTD/TPI group [8]. This prolonged survival with FTD/TPI + BEV was observed in all prespecified subgroups. For example, in patients with *RAS* mutant CRC, mOS was 10.6 months with FTD/TPI + BEV compared with 7.5 months in the FTD/TPI group (HR 0.62, 95% CI 0.48–0.81; Fig. 1) [8]. Additional analyses confirmed that *KRAS* mutations occurring at codon G12 (*KRAS*^G12^) had no impact on mOS and mPFS in SUNLIGHT or on the beneficial effects of FTD/TPI + BEV [22]. Median PFS was also significantly improved in patients receiving FTD/TPI + BEV compared with patients receiving FTD/TPI (HR 0.44, 95% CI 0.36–0.54; *p* < 0.001). PFS at 12 months was 16% in the FTD/TPI + BEV group and 1% in the FTD/TPI group.

The age of the patients had no impact on the benefit of FTD/TPI + BEV compared with FTD/TPI in terms of mOS, mPFS or time to ECOG PS deterioration [23]. There are some data which could suggest that once anti-VEGF treatment is used in a first or second line regimen, it would not be as effective when used again in later line regimens, because resistance towards cancer drugs, including anti-VEGF inhibitors, is a common concern [24, 25]. In SUNLIGHT, clinical benefit was observed regardless of whether patients had received previous treatment with BEV. In patients who had received previous BEV mOS was 9.0 months with FTD/TPI + BEV compared with 7.1 months in the FTD/TPI group (HR 0.72, 95% CI 0.56–0.92; Fig. 1) [8]. The clinical benefit of FTD/TPI + BEV was also observed in subgroups who received any previous anti-VEGF treatment whether this was during the first line regimen only, during the second line regimen only, or in both (Fig. 1) [26]. These findings add to the body of evidence supporting a role for continued inhibition of angiogenesis beyond progression [27, 28].

**Fig. 2:**
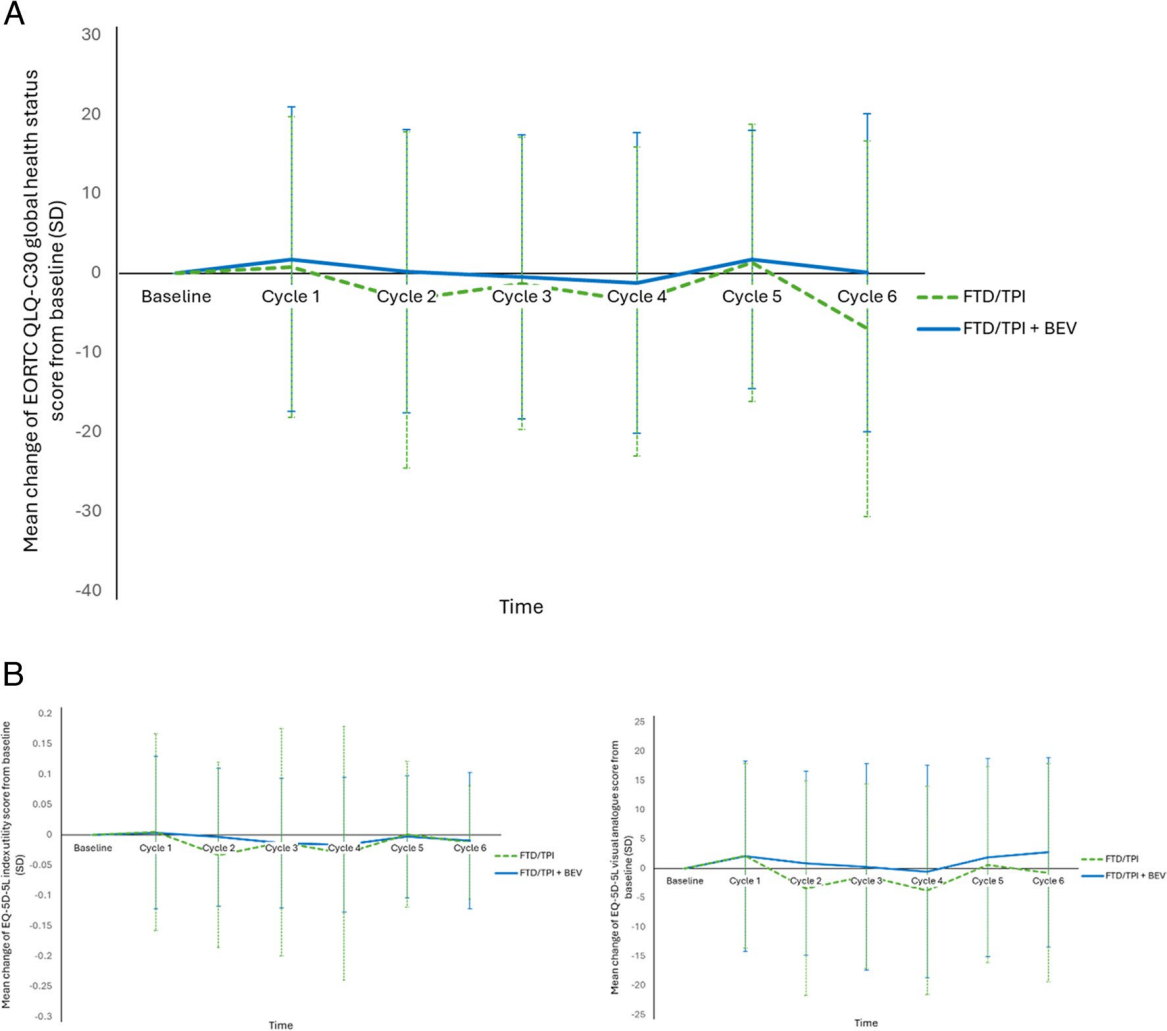
Change from baseline of (**A**) global health status (EORTC QLQ-C30), and (**B**) general QoL (EQ-5D-5L), in the SUNLIGHT trial [29]. Abbreviations: BEV, bevacizumab; EORTC QLQ-C30, The European Organisation for Research and Treatment of Cancer Quality of Life. Questionnaire—Core Questionnaire; EQ-5D-5L, EuroQol 5 Dimension 5 Level; FTD/TPI, trifluridine/tipiracil; QoL, quality of life; SD, standard deviation

Sub-analyses of the SUNLIGHT study determined that the survival benefits of FTD/TPI + BEV as third line treatment regimen of mCRC are associated with maintenance of quality of life (QoL). Health related QoL (HRQoL) was maintained with FTD/TPI + BEV. Both cancer-specific QoL measures (assessed by European Organisation for Research and Treatment of Cancer quality of life questionnaire C30 [EORTC QLQ-C30]), including global health status (GHS; Fig. 2a), functional and symptom scales, and general QoL (assessed by EuroQol 5-Dimension 5-Level questionnaire [EQ-5D-5L]; Fig. 2b), which includes mobility, self-care, usual activities, pain/discomfort and anxiety/depression, and patient’s self-rated health, were maintained [29]. Cancer-specific QoL was maintained for longer in patients receiving FTD/TPI + BEV than in patients receiving FTD/TPI (median time to worsening in GHS [> 10 point change] was 8.5 months versus 4.7 months, respectively [HR 0.50; 95% CI 0.38–0.65]) [29]. Similarly, with the EQ-5D-5L utility score, general HRQoL deteriorated later in patients treated with FTD/TPI + BEV compared with those treated with FTD/TPI [29].

Clinical benefit was observed regardless of baseline ECOG PS score. In patients who had an ECOG PS score of ≥ 1, mOS was 10.8 months with FTD/TPI + BEV compared with 6.3 months in the FTD/TPI group (HR 0.54, 95% CI 0.41–0.73; Fig. 1) [8]. Furthermore, the SUNLIGHT study showed median time to worsening of the ECOG PS score from 0/1 to ≥ 2 was 9.3 months with FTD/TPI + BEV and 6.3 months in the FTD/TPI group (HR 0.54, 95% CI 0.43–0.67) [30].

### Summary of Data on FTD/TPI + BEV as a Third Line Treatment Regimen

The promising activity and manageable safety profile of FTD/TPI + BEV demonstrated in the C-TASK FORCE trial was confirmed in the subsequent trials comparing FTD/TPI + BEV with FTD/TPI. The combination produced significant and clinically relevant improvement in mPFS and mOS compared with FTD/TPI in refractory mCRC [17]. This has since been observed in retrospective studies of patients receiving FTD/TPI + BEV in real-world settings [31, 32]. SUNLIGHT confirmed the efficacy of FTD/TPI + BEV and as a result, FTD/TPI + BEV is now a standard third line regimen in patients with refractory mCRC [2, 8, 9].

A 2023 systematic review and network meta-analysis assessed FTD/TPI + BEV, FTD/TPI, regorafenib, the regorafenib dose-escalation regimen (regorafenib 80 +), fruquintinib, and best supportive care where each treatment was used as a third or later line regimen in patients with mCRC refractory to standard chemotherapy regimens plus anti-VEGF or anti-EGFR therapies. In this review (with the limitation of including studies with different populations with refractory mCRC), FTD/TPI + BEV was the most effective treatment in terms of both OS and PFS among all the options [33]. All treatments assessed had superior median OS compared with best supportive care: FTD/TPI + BEV HR 0.41, 95% credible interval (CrI) 0.32–0.52; FTD/TPI HR 0.67, 95% CrI 0.60–0.76; regorafenib HR 0.71, CrI 0.60–0.84; regorafenib 80 + HR 0.51, 95% CrI 0.32–0.81; and fruquintinib HR 0.65, 95% CrI 0.51–0.83. According to the surface under the cumulative ranking curve methods used, FTD/TPI + BEV had the highest probability of ranking first among these later line therapies [33]. However, findings should be interpreted with caution given that these treatments have not been subject to randomized head-to-head comparative trials. Furthermore, while our narrative review and this previous systematic review focused on efficacy, important differences may exist in the safety profiles of these treatments that will have an impact on treatment decisions. Table 3 summarizes regorafenib and fruquintinib trial data. More recently, findings from the FRESCO-2 study showed that fruquintinib significantly prolonged mOS (7.4 versus 4.8 months for placebo; HR 0·66 (95% CI 0·55–0·80); *p* < 0·0001) and mPFS (3.7 versus 1.8 months for placebo; HR 0·32, 95% CI 0.27–0·39; *p* < 0·0001) compared with best supportive care in 691 patients with refractory mCRC in a later line setting (mostly after ≥ 3 previous lines of therapy including FTD/TPI and/or regorafenib [34].

**Table 3:** Efficacy results of key trials with regorafenib or fruquintinib in patients with refractory mCRC in the third or later line setting [34–38]

	CORRECT		CONCUR		ReDOS		FRESCO-1		FRESCO-2	
Treatment arm	Regorafenib 160 mg/day + BSC	Placebo + BSC	Regorafenib 160 mg/day + BSC	Placebo + BSC	Regorafenib 80–160 mg/day (dose escalation group)	Regorafenib 160 mg/day (standard-dose group)	Fruquintinib 5 mg/day + BSC	Placebo + BSC	Fruquintinib 5 mg/day + BSC	Placebo + BSC
Number of patients	505	255	204	68	54	62	278	138	461	230
OS, median, months (95% CI/IQR)	6.4 (3.6–11.8*)	5.0 (2.8–10.4*)	8.8 (7.3–9.8)	6.3 (4.8–7.6)	9.8 (7.5–11.9)	6.0 (4.9–10.2)	9.3 (8.2–10.5)	6.6 (5.9–8.1)	7.4 (6.7–8.2)	4.8 (4.0–5.8)
PFS, median, months (95% CI/IQR)	1.9 (1.6–3.9)	1.7 (1.4–1.9)	3.2 (2.0–3.7)	1.7 (1.6–1.8)	2.8 (2.0–5.0)	2.0 (1.8–2.8)	3.7 (3.7–4.6)	1.8 (1.8–1.8)	3.7 (3.5–3.8)	1.8 (1.8–1.9)
CR, n (%)	0 (0)	0 (0)	0 (0)	0 (0)	NR	NR	1 (0.4)	0 (0)	0 (0)	0 (0)
PR, n (%)	5 (1)	1 (0.4)	6 (4)	0 (0)	NR	NR	12 (4.3)	0 (0)	7 (2)	0 (0)
SD, n (%)	NR	NR	NR	NR	NR	NR	NR	NR	249 (54)	37 (16)
ORR, n (%)	5 (1)	1 (0.4)	6 (4)	0 (0)	NR	NR	13 (4.7)	0 (0)	7 (2)	0 (0)
DCR, n (%)	207 (41)	38 (15)	70 (51)	5 (7)	NR	NR	NR (62.2)	NR (12.3)	256 (56)	37 (16)

^*^Reported as IQR. Abbreviations: *BSC* best supportive care, *CR* complete response, *DCR* disease control rate, *IQR* interquartile range, *mCRC* metastatic colorectal cancer, *NR* not reported, *ORR* objective response rate, *OS* overall survival, *PFS* progression free survival, *PR* partial response, *SD* stable disease

The maintenance of QoL and ECOG PS observed with FTD/TPI + BEV is of particular importance as prolonging physical performance and controlling symptoms may allow patients to maintain their physical function, and therefore, receive further benefit from subsequent therapy during the continuum of care. This observation makes this combination beneficial for a wide range of patients including vulnerable patients, which was also confirmed in a retrospective study [39]. The efficacy of FTD/TPI + BEV in patients who have previously received different treatment regimens, including those who have received previous anti-VEGF treatments, enhances the possibility of using FTD/TPI + BEV. Other third line treatment regimens for mCRC, such as anti-EGFR rechallenge (limited to *RAS* wild type), have less robust evidence to support their use, and currently it is unknown if such regimens could be reliably effective in as wide a range of patients as FTD/TPI + BEV.

## FTD/TPI + BEV as a First Line Regimen

Key efficacy data regarding FTD/TPI + BEV as a first line regimen are presented in Table 2. For patients with impaired tolerance to aggressive initial therapy, the NCCN guidelines recommend 5-FU/LV or CAP with or without BEV as an option [2]. However, certain adverse effects of CAP + BEV may have a high impact on patient QoL and may be difficult to manage, such as hand foot syndrome [40, 41]. As a result, investigation into other possible therapies was needed.

### The TASCO-1 Trial Data of FTD/TPI + BEV

TASCO-1 was a phase 2 multinational trial conducted to assess FTD/TPI + BEV and CAP + BEV as a first line treatment regimen in patients who were not eligible for intensive therapy [18, 20]. 77 patients received FTD/TPI + BEV and 76 received CAP + BEV. Patients assigned to FTD/TPI + BEV received FTD/TPI (35 mg/m^2^) orally twice daily, 5 days a week (plus 2 days of rest) for 2 weeks in a 28-day cycle, and BEV (5 mg/kg) was administered intravenously every 2 weeks (on days 1 and 15 of each cycle).

There was a trend for longer mOS in patients receiving FTD/TPI + BEV than patients receiving CAP + BEV (HR 0.78, 95% CI 0.55–1.10), and for longer mPFS with FTD/TPI + BEV than with CAP + BEV (HR 0.71, 95% CI 0.48–1.06; Table 2) [18, 20]. In some subgroups, mOS with FTD/TPI + BEV may have been longer than with CAP + BEV – for example, patients with a *RAS* mutation (HR 0.81, 95% CI 0.52–1.26), and patients with an ECOG PS score of 2 (HR 0.50, 95% CI 0.20–1.26). The QLQ-C30 questionnaire showed no clinically relevant changes from baseline in the GHS and functional scales, and in most symptom scales. The results from TASCO-1 provided the rationale for a larger-scale phase 3 trial.

### The Pivotal Phase 3 Trial, SOLSTICE

SOLSTICE was a phase 3 international trial in patients with mCRC not eligible for intensive full-dose doublet or triplet chemotherapy and was designed to have similar inclusion criteria for patients as in TASCO-1 [18–20]. FTD/TPI + BEV or CAP + BEV were used as the first line treatment regimen. 426 patients received FTD/TPI + BEV and 430 received CAP + BEV. The primary endpoint was PFS, and after a median follow-up of 16.6 months the HR for median PFS with FTD/TPI + BEV versus CAP + BEV was 0·87 (95% CI 0·75–1·02; *p* = 0·0464 [protocol-defined significance level of *p* = 0·021]; Table 2), and thus, superiority of FTD/TPI + BEV over CAP + BEV was not observed. mOS was not observed to be superior in patients receiving FTD/TPI + BEV compared to patients receiving CAP + BEV (19.7 months and 18.6 months, respectively, HR 1.06, 95% CI 0.90–1.25; Table 2) [19, 21].

**Fig. 3:**
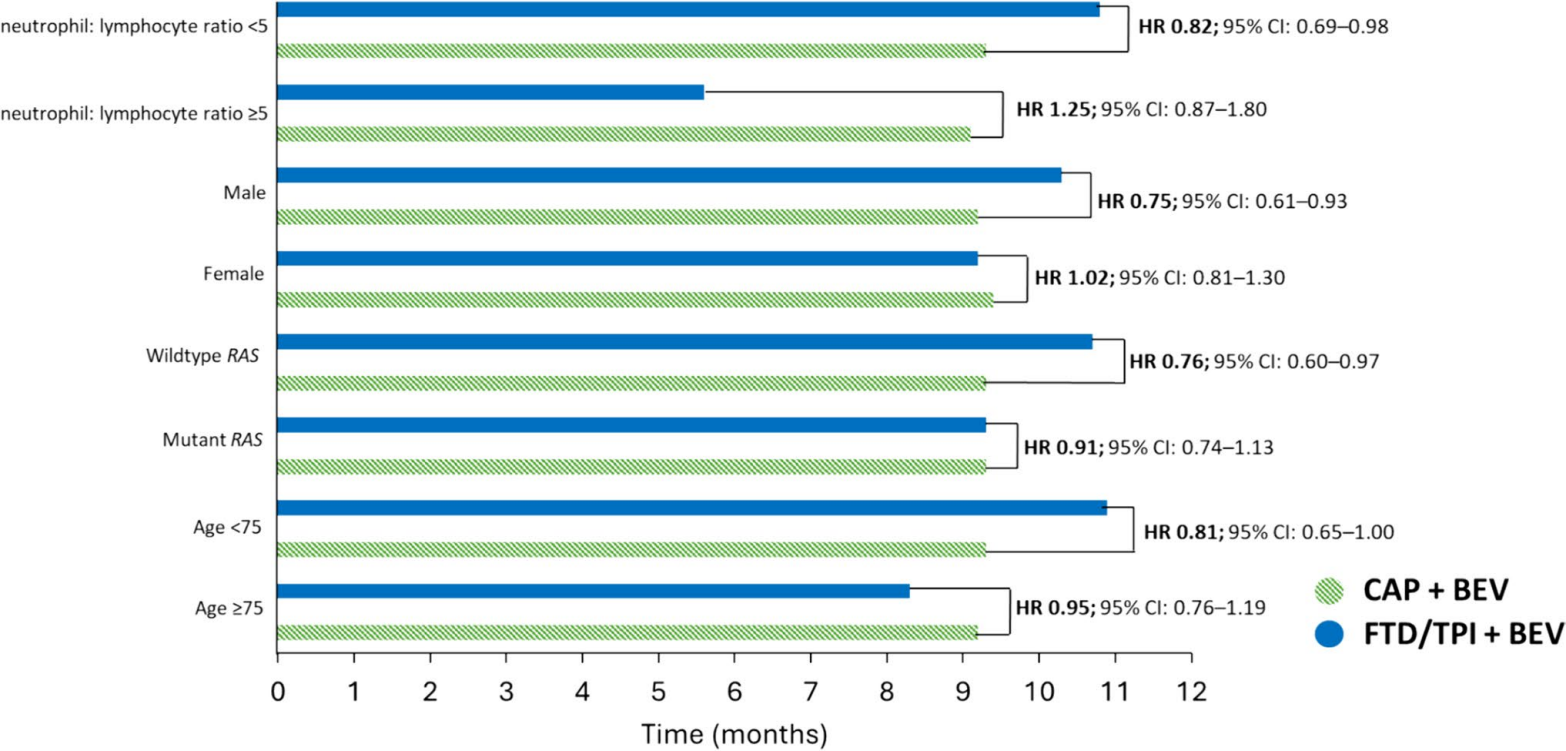
Median progression-free survival in SOLSTICE subgroups [19]. Abbreviations: BEV, bevacizumab; CAP, capecitabine; CI, confidence interval; FTD/TPI, trifluridine/tipiracil; HR, hazard ratio

In a subgroup analysis, the HR for PFS was consistent with that of the intention-to-treat population for most subgroups. Three patient subsets were associated with improved mPFS outcomes with FTD/TPI + BEV than with CAP + BEV: *RAS* wildtype, male gender, and neutrophil:lymphocyte ratio < 5 (Fig. 3) [19]. For most subgroups, FTD/TPI + BEV performed with similar efficacy to CAP + BEV, including in patients aged < 75 (HR 0.81, 95% CI 0.65–1.00) and patients aged ≥ 75 (HR 0.95, 95% CI 0.76–1.19) [19]. In HRQoL assessments, mean baseline GHS in the QLQ-C30 dataset (*n* = 366 in both groups) were similar in both groups and showed no clinically relevant change from baseline (i.e., increase or decrease of > 10 points in the GHS) at any time point up to week 60 [19].

### Summary of Data on FTD/TPI + BEV as a First Line Regimen

Following promising results in the TASCO-1 trial as a first line regimen for unresectable mCRC, the failure of FTD/TPI + BEV to meet the primary endpoint in SOLSTICE could be viewed as a negative result. However, in SOLSTICE, FTD/TPI + BEV had a similar effect compared to CAP + BEV in terms of PFS, OS, and DCR. In addition, FTD/TPI + BEV had no clinically relevant impact on the QoL compared with CAP + BEV. In this setting, FTD/TPI + BEV is therefore a valuable alternative to CAP + BEV. If, for example, specific adverse events associated with the CAP + BEV regimen, such as hand foot syndrome, are a particular issue, then FTD/TPI + BEV is an equally effective alternative with a different safety profile. Another example is the potential use of FTD/TPI + BEV in patients with CAP contraindication due to dihydropyrimidine dehydrogenase deficiency. A factor to consider is that CAP + BEV performed better in SOLSTICE than in TASCO-1, whereas FTD/TPI + BEV performance remained the same. The reasons for this are unclear. When considering the additional observation that there is no extra deleterious effect on QoL observed with FTD/TPI + BEV compared to CAP + BEV, FTD/TPI + BEV represents a feasible alternative option as a first treatment for mCRC when doublet or triplet chemotherapy regimens are not suitable.

## Conclusions/Discussion

This narrative review provides an overview of the clinical trial data on FTD/TPI + BEV in patients with mCRC. Among patients with refractory mCRC, the phase 3 SUNLIGHT trial showed longer survival with treatment with FTD/TPI + BEV versus FTD/TPI, irrespective of previous anti-VEGF therapy, *RAS* mutation status, and age, and is considered by guidelines as a standard of care in third line [2, 9]. In the first line SOLSTICE study, FTD/TPI + BEV had similar efficacy to CAP + BEV irrespective of *RAS* mutation status and age. FTD/TPI + BEV should be considered a suitable alternative as an initial treatment of mCRC, particularly in cases where the patient or clinician wants to limit the risk of specific CAP-associated adverse events such as hand foot syndrome, or when there are CAP contraindications. Additional integrated analyses in the form of a meta-analysis may provide more evidence of treatment effect to support these summarized findings.

## Key References

- Prager GW, Taieb J, Fakih M, Ciardiello F, Van Cutsem E, Elez E, et al. Trifluridine-tipiracil and bevacizumab in refractory metastatic colorectal cancer. N Engl J Med. 2023;388(18):1657–67. 10.1056/NEJMoa2214963. 
  - This reference is of outstanding importance as it reports on the pivotal phase 3 SUNLIGHT trial that established trifluridine/tipiracil + bevacizumab as the recomended third-line treatment for metastatic colorectal cancer.
- Gao L, Tang L, Hu Z, Peng J, Li X, Liu B. Comparison of the efficacy and safety of third-line treatments for metastatic colorectal cancer: a systematic review and network meta-analysis. Front Oncol. 2023;13:1269203. 10.3389/fonc.2023.1269203. 
  - This reference is of importance as this review assessed all third or later line treatment options for metastatic colorectal cancer and established trifluridine/tipiracil + bevacizumab as the most effective treatment in terms of overall survival and progression-free survival.
- Andre T, Falcone A, Shparyk Y, Moiseenko F, Polo-Marques E, Csoszi T, et al. Trifluridine-tipiracil plus bevacizumab versus capecitabine plus bevacizumab as first-line treatment for patients with metastatic colorectal cancer ineligible for intensive therapy (SOLSTICE): a randomised, open-label phase 3 study. Lancet Gastroenterol Hepatol. 2023;8(2):133-44. 10.1016/S2468-1253(22)00334-X. 
  - This reference is of outstanding importance because it is a large phase 3 study that suggests trifluridine/tipiracil + bevacizumab is a suitable alternative to capeticibine + bevazisumab as first line treatment of metastatic colorectal cancer.

## Data Availability

No datasets were generated or analysed during the current study.
